# Affordable pediatric CD4 counting by flow cytometry in Malawi

**DOI:** 10.1002/cyto.b.20411

**Published:** 2008-02-28

**Authors:** Calman A MacLennan, Felix Dzumani, Alinane Namarika, Peter Moons, Edward Senga, Malcolm E Molyneux, Mark T Drayson, James E G Bunn

**Affiliations:** 1 Malawi-Liverpool-Wellcome Trust Clinical Research Programme, College of Medicine, University of MalawiBlantyre, Malawi; 2 Medical Research Council Centre for Immune Regulation and Clinical Immunology Service, Division of Immunity and Infection, University of BirminghamBirmingham, United Kingdom; 3 Division of Medical Microbiology, School of Infection and Host Defence, University of LiverpoolUnited Kingdom; 4 Department of Microbiology, College of Medicine, University of MalawiBlantyre, Malawi; 5 Department of Biochemistry, College of Medicine, University of MalawiBlantyre, Malawi; 6 Department of Pediatrics, College of Medicine, University of MalawiBlantyre, Malawi; 7 Department of Community Health, College of Medicine, University of MalawiBlantyre, Malawi

**Keywords:** CD4 lymphocyte count, flow cytometry, Malawi, HIV, pediatrics

## Abstract

**Background:**

Rapid expansion of antiretroviral therapy in Malawi has occurred in the relative absence of suitable pediatric CD4 counting facilities. We have recently validated in adults a simplified affordable flow cytometric CD4 counting method, the Blantyre count. There is a need for this technology to transfer to government laboratories run by local staff, and to be validated in children, where %CD4/lymphocyte values are required.

**Methods:**

We assessed agreement of %CD4/lymphocyte values determined by the Blantyre count and Panleucogate methods on an EPICS XL-MCL flow cytometer on 113 venous blood samples from HIV-seropositive children in Blantyre, Malawi. All assays were performed by two Malawian laboratory technicians.

**Results:**

Overall bias between the two methods was −0.13% (95% CI −0.37 to 0.11) and limits of agreement were −2.69 to 2.43% (95% CI −3.11 to −2.27 and 2.01 to 2.85). Limits of agreement were within −3.00 and 3.00 for each laboratory technician. Coefficient of variation for the Blantyre count assay was 2.0% and samples showed good stability over 5 days.

**Conclusions:**

The Blantyre count method can accurately determine %CD4/lymphocyte values in blood of HIV-seropositive children on an EPIC XL-MCL flow cytometer at a reagent cost of US $0.21 per test or less. The assay can be competently carried out by local laboratory technicians.

The facility of flow cytometry to rapidly analyze the surface characteristics of cells makes it ideally suited for determining relative levels of cell populations within a blood sample. For this reason, flow cytometry is the gold-standard platform for measuring CD4 cells as a percentage of total lymphocyte count (%CD4/lymphocyte) as required for the immunological staging and monitoring of HIV-infected children below 5 years of age (1,2).

Antiretroviral therapy for the treatment of HIV/AIDS became freely available at the point of delivery in government clinics in Malawi in 2004. Since then, there has been a rapid scaling-up in the provision of antiretroviral therapy for both children and adults. By the end of June 2007 in public sector clinics, 110,075 individuals had been commenced on treatment, of whom 8% are children aged 14 years and below (3).

The decision to start an HIV-infected individual on antiretroviral therapy in Malawi has been largely based on clinical staging criteria so that those with WHO stage III and IV HIV/AIDS are commenced on therapy, whereas those with stage I and II disease are not. We have previously shown the limitations of this approach when used among adult Malawians presenting to the antiretroviral therapy clinic at Queen Elizabeth Central Hospital, Blantyre. Thirty-eight percent of new adult patients attending the antiretroviral therapy clinic with clinical Stage I and II disease were severely immunosuppressed with CD4 counts <200 cells/μl, while 20% with Stage III and IV disease had CD4 counts >350 cells/μl (4). In Malawian children, there are similar limitations in using WHO clinical staging criteria, particularly in a country where 48% of below 5 years old are stunted, 22% are underweight, and 5% are wasted. These clinical features form part of the WHO staging criteria, but are predominantly due to factors other than HIV in Malawi (5).

Where CD4 counts have been available in Malawi, both at Queen Elizabeth Central Hospital and elsewhere, these tests have mostly been performed using FACSCount (Becton Dickinson) dedicated CD4 counting instruments (6). The FACSCount provides absolute CD3, CD4, and CD8 counts, but is currently unable to generate CD4 counts as a percentage of the total lymphocyte count (%CD4/lymphocyte) without obtaining a separate total lymphocyte count from a hematological analyzer. The %CD4/lymphocyte can then be calculated by dividing the CD4 count from the FACSCount instrument with the total lymphocyte count from the hematological analyzer.

Such a “reversed dual-platform” approach is unnecessarily cumbersome and expensive as it requires the use of two instruments, when even the most basic flow cytometers can generate %CD4/lymphocyte values on a single platform. Importantly, we have shown that this reversed dual-platform approach is inherently inaccurate when compared with established single-platform flow cytometric CD4 counting. Comparing %CD4/lymphocyte values obtained using FACSCount and a hematological analyzer with those obtained using Multitest with TruCount tubes (TruCount assay, Becton Dickinson) on a FACSCalibur flow cytometer (Becton Dickinson), we found unacceptably wide limits of agreement (−5.83 to 7.66%, 95% CI −6.87 to −4.79 and 6.62 to 8.70) (4). (A modification of the FACSCount system that will determine %CD4/lymphocyte values has recently been tested (7) and should become available in due course.)

The CyFlow Counter (Partec) has been proposed as an alternative dedicated CD4 counting instrument for Malawi and has been used by Medécins Sans Frontières in this country. Information is not available concerning its use in %CD4/lymphocyte determination in children in Malawi. However, a study comparing accuracy of the CyFlow Counter with FACSCount for producing absolute CD4 counts at Chiradzulu District Hospital in Malawi found poor limits of agreement between the two assays that were greater than −150 to 150 cells/μl (8).

In the past few years, a small number of larger flow cytometers have appeared in Malawi, principally in research settings, but more recently in regional government hospitals, nongovernmental organization clinics, and in a laboratory of the national medical school. These have provided the opportunity to use a single-platform approach for determining both absolute CD4 counts and %CD4/lymphocyte.

TruCount and Panleucogate (Beckman Coulter) (9) commercial kit-based flow cytometric CD4 counting assays for use on these larger cytometer platforms are available in Malawi for US $5–6 reagent cost per test. These prices represent a significant saving on the price of such kits in developed countries, but are still too expensive for the widespread introduction of flow cytometric CD4 counting in Malawi, especially when considering the cost of annual maintenance contracts of approximately $12,000 for each flow cytometer.

In South Africa, the successful Panleucogate/EPICS XL-MCL cytometer-based national CD4 counting program (10) provides absolute and percentage CD4 counts at an equivalent reagent price of $3 per test with the manufacturer covering the cost of instrument installation, maintenance, training, and quality assurance. Such a cost-effective agreement is possible because of the large number of predicted CD4 assays per year country-wide. Only five EPICS cytometers are currently sited in Malawi, and there is no unified national arrangement on CD4 counting.

To offset the high capital outlay of flow cytometers and their annual maintenance programs in Malawi, we have recently optimized and validated an accurate and affordable method of flow cytometric CD4 counting, the Blantyre count, on a FACSCalibur flow cytometer at the Malawi-Liverpool-Wellcome Trust Clinical Research Programme. Absolute CD4 and %CD4/lymphocyte enumeration are performed at a reagent cost of $0.44, which is reduced to $0.11 if only %CD4/lymphocyte is required, by using a CD4/CD45 antibody combination with reduced volumes of blood and reagents (4).

The Blantyre count technology has recently been transferred from a research laboratory to the EPICS XL-MCL flow cytometer (Beckman Coulter) at the College of Medicine in Blantyre. In this study we assessed the agreement of this method for affordable %CD4/lymphocyte determination with the Panleucogate method using pediatric venous blood samples. All flow cytometric operations were performed by Malawian laboratory technicians.

## MATERIALS AND METHODS

### Setting

Blantyre is the largest city in Malawi and is the principal city in the Southern Region of the country. The College of Medicine, University of Malawi, is the only medical school in Malawi and is situated adjacent to Queen Elizabeth Central Hospital (QECH), the largest hospital in Malawi. The hospital has busy adult and pediatric antiretroviral clinics, as well as inpatient facilities.

### Instrumentation

Flow cytometric CD4 counting was performed using an EPICS XL-MCL flow cytometer (Beckman Coulter) in the Department of Biochemistry, College of Medicine. This instrument came into clinical service in 2005. Internal quality control is performed daily on the flow cytometer using Immuno-Trol cells (Beckman Coulter).

### Blood Samples

All blood samples used in the study were EDTA-anticoagulated blood from HIV-seropositive children. Samples were sent to the laboratory from QECH and health centers in Blantyre for CD4-positive lymphocyte determination. All samples used in the repeatability, stability, and assay comparison studies were venous blood samples.

During the course of the study, we published a validation in adults of the use of finger-prick blood samples for CD4-positive lymphocyte enumeration (11) and subsequently began to use finger-prick blood samples clinically for %CD4/lymphocyte determination in children where attempts to obtain a venous blood sample had been unsuccessful. Approximately, 10 samples were taken by this method. These were not included among the 113 samples in the Blantyre count/Panleucogate agreement study.

### Assay Operators

Both index test and reference standard flow cytometric assays were performed on each sample by one of the two Malawian laboratory technicians, both holding Diplomas in Laboratory Technology and one with a BTech in Biomedical Technology. Both technicians had experience of performing the Panleucogate CD4-counting assay (PLG, Beckman Coulter) (9), but no other flow cytometric assays. Both technicians had attended a 1-week training course in the general operation of the EPICS XL-MCL flow cytometer arranged locally by Beckman Coulter. They received a half-day of training in the Blantyre count method from Dr. C. A. MacLennan in the College of Medicine flow cytometry laboratory in Blantyre.

### Reference Standard (Panleucogate Assay)

The Panleucogate method was used as the reference standard, because it is a robust established commercial flow-cytometric CD4 counting assay that determines %CD4/lymphocyte values as well as absolute CD4 counts, and is recommended for use on Beckman Coulter EPICS flow cytometers, and was developed and is extensively used in South Africa (9,10). Panleucogate assays were performed according to the manufacturer's instructions (12). One hundred microliters of EDTA-anticoagulated venous blood was mixed with 10 μl of CD45-FITC/CD4-PE antibody (FlowCare PLG CD4 Reagent, Beckman Coulter) and incubated for 20 min at room temperature in the dark. Red cells were then lysed using the ImmunoPrep Reagent System and automated TQ-Prep Workstation (both Beckman Coulter). Data acquisition and analysis were performed on the EPICS XL-MCL flow cytometer using the Panleucogate gating strategy and System II Software Version 3.0 (Beckman Coulter).

**Fig. 1:**
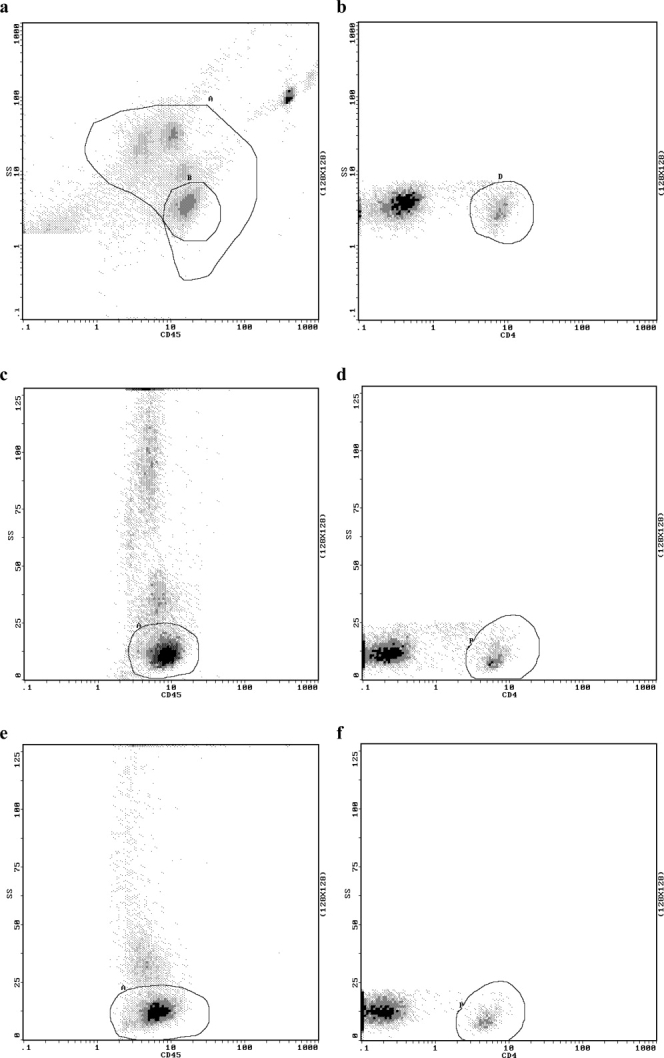
Panleucogate and Blantyre count gating strategies for %CD4/lymphocyte determination using System II Software. (**a** and **b**) Panleucogate strategy on venous blood sample with (a) CD45(log)/sidescatter(log) dot plot showing total leucocytes gate (the Panleucogate) (A) and total lymphocytes (B), (b) CD4(log)/sidescatter(log) dot plot of total lymphocytes (A) showing CD4-positive lymphocytes (D). (**c** and **d**) Blantyre count strategy on venous blood sample with (c) CD45(log)/sidescatter(linear) dot plot showing total lymphocytes (A), (d) CD4(log)/sidescatter(linear) dot plot of total lymphocytes (A) showing CD4-positive lymphocytes (B). Same venous blood sample as used in (a) and (b). (**e** and **f**) Blantyre count strategy on finger-prick blood sample with (e) CD45(log)/sidescatter(linear) dot plot showing total lymphocyte gate (A), (f) CD4(log)/sidescatter(linear) dot plot of total lymphocytes showing CD4-positive lymphocytes (B). %CD4/lymphocyte values are calculated directly from the number of cells in the CD4-positive lymphocyte region and number of cells in the total lymphocyte region. SS, side-scattered light.

With the Panleucogate gating strategy (9,10), 5,000 lymphocytes are acquired per sample with threshold for acquisition set on the forward scatter (size) channel. Total leucocytes (the panleucogate) and total lymphocytes are first gated using a CD45-FITC (logarithmic scale) against side scatter (logarithmic scale) dot plot (Fig. 1a). CD4-positive lymphocytes are then determined by gating the CD4-bright population using a CD4-PE (logarithmic scale) against side-scatter (logarithmic scale) dot plot of the total lymphocyte population (Fig. 1b). %CD4/lymphocyte is determined from the CD4-positive lymphocyte and total lymphocyte values.

CD4 counting at the College of Medicine using the Panleucogate assay has recently been enrolled for external quality assurance monitoring with UK NEQAS (13). Testing the six most recent samples distributed by UK NEQAS gave a score of 11 out of a maximum possible 12 for the %CD4/lymphocyte parameter: %CD4/lymphocyte values for 5/6 samples were within 1 standard deviation and 1/6 samples was within 2 standard deviations of the UK NEQAS value.

### Index Test (Blantyre Count Assay)

The Blantyre count method for %CD4/lymphocyte determination was essentially the Blantyre count (percentage) variant described previously (4) with modifications for using pediatric blood samples and the EPICS XL-MCL flow cytometer. These modifications involved increasing the volume of the two antibodies used from 0.5 to 1.0 μl each and establishing the Blantyre count gating strategy on the System II Software supplied with the EPICS XL-MCL flow cytometer. We previously validated the use of 0.5 μl of each antibody as twice the minimum amount required to distinguish CD4-positive and total lymphocytes as discrete populations in adult blood. The amount of antibody used in the present study was increased because the blood lymphocyte counts in children are higher than in adults. The actual antibodies and red cell lysing fluid used were the same as those in the previously published study (4). The Blantyre count method is a noncommercial method and although similar to the Panleucogate method, differs from it in the following respects: smaller volumes of blood and antibodies are used, red cell lysis is performed without the use of an automated workstation, threshold for acquisition is set on the FL1 (FITC) channel rather than forward light scatter, and a linear rather than logarithmic side light scatter scale is used in the gating strategy.

One microliter of phycoerythrin-conjugated anti-human CD4 antibody (CD4-PE) and 1 μl of fluorescein isothiocyanate-conjugated anti-human CD45 antibody (CD45-FITC) (both Becton Dickinson) were mixed with 20 μl of EDTA-anticoagulated venous blood and incubated in the dark at room temperature for 15 min. One hundred eighty microliters of 1× FACS lysing solution (Becton Dickinson) was then added to lyse red cells and the solution was further incubated for 10 min at room temperature in the dark prior to acquisition and analysis of data on the flow cytometer using the Blantyre count gating strategy and System II Software. Pipetman P20 and P200 pipettes (Gilson, France) were used for all pipetting steps. The Blantyre count gating strategy has been described previously (4). Five thousand lymphocyte events are acquired per sample with threshold for acquisition set on the FL1 (FITC) channel. Total lymphocytes for each acquisition are determined by gating the side scatter-low CD45-bright population using a CD45-FITC (logarithmic scale) against side scatter (linear scale) dot plot (Fig. 1c). CD4-positive lymphocytes are determined by gating the CD4-bright population using a CD4-PE (logarithmic scale) against side-scatter (linear scale) dot plot of the total lymphocyte population (Fig. 1d). %CD4/lymphocyte values were determined from these two values.

### Repeatability, Stability, and Interoperator Variability

Repeatability was assessed by performing five repeats of the Blantyre count assay on six fresh blood samples. Stability was assessed by performing five repeats of the assay daily on two blood samples for 5 days. The blood samples were kept at room temperature throughout the 5-day period.

To estimate interoperator variability in the sample preparation stages (pipetting, antibody labeling, and red cell lysis) and confirm ease of use of the Blantyre count method, two groups of students (*n* = 9 and 8) in the second year of their Diploma in Laboratory Technology course at the University of Malawi performed the Blantyre count assay following a standard operating procedure. The students had no prior experience of the assay.

### Assay Comparison Study

One hundred thirteen consecutive pediatric venous blood samples received in the laboratory for CD4 determination between March 14, 2007 and May 4, 2007 were assayed for %CD4/lymphocyte using both Panleucogate and Blantyre count assays within an hour of each other. All samples were from HIV-seropositive children. HIV-seropositive children under 18 months presenting with a clinical illness, potentially HIV-related, were included, but confirmatory PCR diagnosis was not available. Samples were rejected if clotted or incorrectly labeled. There were no other inclusion or exclusion criteria. Samples were assayed within 24 h of venesection. Data collection was planned before the assays were performed. All blood samples from all participants underwent the index and reference standard tests. No adverse events occurred from performing these tests. Analysis was performed blinded to the results of the paired assay. No data were missing and all data were used. No cut-offs were applied to the results.

### Statistical Analysis

Agreement between methods was examined using Stata 9 by estimating bias and limits of agreement (bias ± 1.96 SD) with 95% confidence intervals as described by Bland and Altman (14). Repeatability was assessed using coefficients of variation obtained from five repeats of assays.

## RESULTS

### Characteristics of Patients

All blood samples were from HIV-seropositive children (51% male) attending HIV staging and treatment clinics at Queen Elizabeth Central Hospital and health centers in Blantyre. Twenty-six children were less than 18 months old and median age of all children was 38 months. Most children were ambulatory and attending outpatient services, with 14% of samples taken from inpatients. Twenty-two percent of children were recovering from severe acute malnutrition. Clinical staging was by WHO criteria (2), and was available for 63 children. Of these, 33% were stage I or II, and 67% stage III or IV or presumptive diagnosis (if under 18 months). Immunological staging of all children using WHO age-specific %CD4/lymphocyte cut-offs (2) found that 65% were severely immunosuppressed. Only two children were currently on antiretroviral therapy. Children meeting clinical or immunological staging criteria for starting antiretroviral therapy were referred for this.

### %CD4/lymphocyte Values in Agreement Studies

**Fig. 2:**
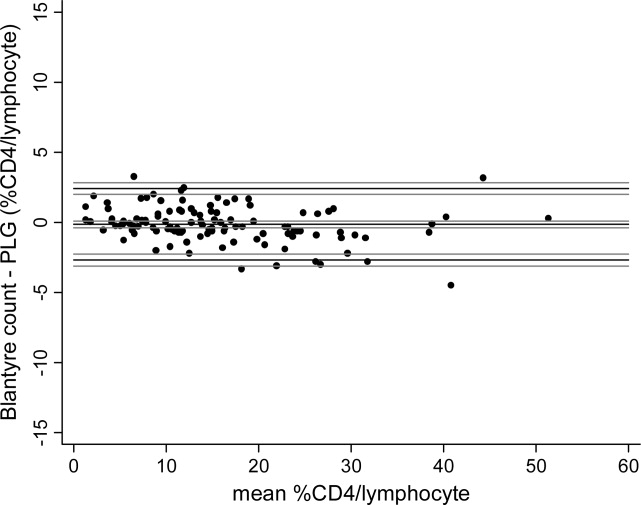
Comparison of CD4 counts determined as a percentage of total lymphocyte count (%CD4/lymphocyte) using the Blantyre count and Panleucogate methods with 113 venous blood samples from HIV-seropositive Malawian children. Mean %CD4/lymphocyte values are the average of the %CD4/lymphocyte values generated by the Blantyre count and Panleucogate methods for each sample. Black lines depict bias and upper and lower limits of agreement. Gray lines denote 95% confidence intervals for these values.

The median %CD4/lymphocyte value by the Panleucogate method was 13.7% (mean 9.51%, range 0.73–51.2%). The median %CD4/lymphocyte value by the Blantyre count method was 13.8% (mean 9.75%, range 1.42–51.5%). Agreement between %CD4/lymphocyte values obtained using the two methods was excellent with a mean difference (bias) of −0.13% (CI −0.37 to 0.11) and limits of agreement −2.69 to 2.43% (CI −3.11 to −2.27 and 2.01 to 2.85) (Fig. 2, Table 1). When data were analyzed according to assay operator, similar bias and limits of agreement were obtained for both laboratory technicians with biases of −0.17 and −0.07% and limits of agreement within −3.00 and 3.00 (Table 1).

**Table 1:** Estimated Bias and Limits of Agreement, with 95% Confidence Intervals for Percentage CD4 Cell Counts (%CD4/lymphocyte) Determined by Blantyre Count and Panleucogate Assays on 113 Venous Blood Samples from HIV-Seropositive Malawian Children. Overall Values and Values for Each Laboratory Technician Are Given

				Limits of agreement			
	Samples	Bias	95% Confidence interval	Lower limit	95% Confidence interval	Upper limit	95% Confidence interval
Overall	113	−0.13	(−0.37 to 0.11)	−2.69	(−3.11 to −2.27)	2.43	(2.01 to 2.85)
Technician 1	67	−0.17	(−0.46 to 0.12)	−2.48	(−2.98 to −1.98)	2.14	(1.64 to 2.64)
Technician 2	46	−0.07	(−0.51 to 0.37)	−2.98	(−3.74 to −2.21)	2.83	(2.07 to 3.59)

### Repeatability Study

Mean %CD4/lymphocyte values for each of the six blood samples tested were 15.8 to 30.0%. Mean coefficient of variation for the Blantyre count assay was 2.0% (range 0.9–3.5%). There was no association between %CD4/lymphocyte value and coefficient of variation. These coefficients of variation are similar to the published values from the manufacturer for the Panleucogate method (1.67% using control cells with a %CD4/lymphocyte of 48.1%; 3.18% using control cells with a %CD4/lymphocyte of 22.0%) (12).

### Stability Study

For the duration of the stability study, total lymphocytes and CD4-positive lymphocyte populations could be clearly distinguished. There was a gradual increase in %CD4/lymphocyte of the two samples tested such that the day 5 values were 2.6 and 2.3% higher than the day 1 values. Although coefficients of variation were lowest for the day 1 values, there was no clear trend and these ranged from 1.6 to 6.1%.

### Interoperator Variability Study

Coefficients of variation for %CD4/lymphocyte values obtained by each group of students using one blood sample in each group were 2.5 and 0.8%. The mean values obtained by each group were within 0.3% of the value obtained by the class instructor.

### Finger-Prick Blood Samples

Following on from our recent validation of finger-prick blood samples for CD4-lymphocyte determination in adults (11), we encountered no difficulties in labeling and gating CD4-lymphocyte and total lymphocyte populations for the determination of %CD4/lymphocyte values in finger-prick samples taken from children using the Blantyre count method with the EPICS XL-MCL flow cytometer (Figs. 1e and 1f).

## DISCUSSION

The Blantyre count method was originally developed as a simplified affordable method of CD4 counting using a Becton Dickinson flow cytometer and reagents, on blood samples from HIV-infected Malawian adults (4). In the present study, we demonstrate the successful transfer of this technology in its simplest form with only minimal modifications (increased antibody quantities and Blantyre count gating strategy on System II Software) for %CD4/lymphocyte determination in venous blood from HIV-infected Malawian children using a Beckman Coulter flow cytometer.

The rationale for the present study was to provide accurate low cost %CD4/lymphocyte values for the Department of Pediatrics at Queen Elizabeth Central Hospital where previously only the reversed dual-platform FACSCount approach had been available. The minimal bias and narrow limits of agreement obtained when comparing the Blantyre count and Panleucogate methods in the present study indicate the accuracy of Blantyre count for %CD4/lymphocyte determination in pediatric blood samples on a Beckman Coulter flow cytometer.

Of equal significance is the low cost of reagents associated with this approach. Using locally available prices, the reagent costs per Blantyre count %CD4/lymphocyte assay are $0.21 which represents a 96% cost-reduction on the best locally available price of the Panleucogate and FACSCount assays (both $6 per test). The Blantyre count reagent cost is greater than that given previously (4), because we used twice the amount of antibodies compared with the original Blantyre count study. However, while performing this study, we have reduced the reagent cost further by using generic monoclonal antibodies in the Blantyre count assay as has been described previously (15).

Whereas all flow cytometric operations in the original Blantyre count study were performed by scientists holding PhDs in immunology and with considerable experience of flow cytometry, all experimental procedures in the present study were performed by laboratory technicians with minimal flow cytometry training. This indicates that the Blantyre count methodology is appropriate for use by local staff in Malawi, given appropriate technical training and support.

Where highly skilled laboratory staff and accurately calibrated pipettes may not be available (16), the Blantyre count technique has the advantage that precise pipetting accuracy is not critical for %CD4/lymphocyte determination, since the CD4+ T-lymphocyte number is measured as a ratio of the total lymphocyte number in the same tube of blood. Use of this cell ratio is probably the reason for the low coefficients of variation both in the repeatability study and in the interoperator variability study with student laboratory technicians. It may also account for the relative stability of %CD4/lymphocyte values in aged blood samples, since loss of lymphocytes with time is likely to affect both CD4-positive and CD4-negative lymphocytes to similar degrees.

This lack of reliance on accurate pipetting is in contrast to absolute CD4-positive lymphocyte enumeration on the majority of single platform flow cytometer operations as well as many nonflow cytometric methods, where use of precise blood and counting bead volumes is essential. In addition, any strategy that relies on first generating an absolute CD4 count and then deriving the percentage value from a total lymphocyte count is likely to suffer the same pitfall as using this “reversed-dual platform” approach with the FACSCount instrument. Thus even with the possible advent of CD4 “rapid tests” (17) for absolute CD4 counts in the future, the flow cytometer should retain its position as the methodology of choice for CD4 percentage determination.

Compared with the Blantyre count method previously published, we doubled the quantities of CD4-PE and CD45-FITC antibody used per test, in view of the higher number of lymphocytes in pediatric compared with adult blood samples. This was to ensure adequate labeling to distinguish total lymphocytes and the CD4-positive lymphocyte subset. Otherwise our sample preparation was identical to that described previously (4). We continued to use FACS lysing solution (Becton Dickinson) for red cell lysis and fixation rather than the ImmunoPrep Reagent system and TQ-Prep Workstation (Beckman Coulter) as recommended by the manufacturer of the Panleucogate method. This suggests that for %CD4/lymphocyte determination on the EPICS instrument, use of the Coulter TQ-Prep Workstation is not necessary.

As with the established Blantyre count method and in contrast with the Panleucogate method, threshold for acquisition was set on the FL1 (CD45-FITC) channel rather than forward light scatter, the side light scatter parameter was set to a linear rather than a log scale to aid discrimination of lymphocytes and monocytes, and a total lymphocyte rather than total leucocyte primary gate was utilized.

We have recently validated the use of finger-prick blood samples for flow cytometric CD4 counting (11), indicating that even in rural settings in Malawi where a trained phlebotomist is not available, %CD4/lymphocyte could be obtained on patients by transporting finger-prick blood samples to the regional centers. Finger-prick blood samples are successfully being used in our clinics for children where venous sampling has not been possible.

Robust external quality assurance of each CD4 counting method used in each laboratory is essential to avoid inaccuracies in CD4 testing. Since its development early in 2006, the Blantyre count method has been registered for external quality assurance with UK NEQAS at the Malawi-Liverpool-Wellcome Trust Clinical Research Programme. The method currently has a maximum score of 12 for %CD4/lymphocyte values indicating that within the last 6 months, %CD4/lymphocyte values on all UK NEQAS samples tested have been within 1 standard deviation of the target value. We are in the process of registering the Blantyre count method with UK NEQAS for %CD4/lymphocyte determination on the EPICS flow cytometer at the College of Medicine.

With the almost negligible reagent costs incurred by using generic antibodies with the Blantyre count method described in this article, attention must now focus on nonreagent costs. These include capital outlay for the flow cytometer, maintenance contracts, and staff salaries and as such are relatively fixed so that the overall cost per assay becomes inversely proportional to the assay throughput. Flow cytometers have the advantage over nonflow cytometric methodologies and the FACSCount instrument in being able to handle over 250 samples a day with ease (18). Therefore, a cost-effective system would aim to operate at this level.

Currently there is no coordinated national program or strategy for CD4 counting in Malawi for either percentage or absolute counts. The advantages and disadvantages of centralized versus decentralized models would need to be considered for such a program taking into account the costs of instruments and their maintenance together with practicalities of sample and/or patient transport and results dissemination.

There are three regional capitals in Malawi: Blantyre (Southern Region), Lilongwe (Central Region), and Mzuzu (Northern region). Each of these centers already has a Beckman Coulter EPICS flow cytometer in place, and so establishment of Blantyre count technology in the remaining two cities is potentially straightforward. Since %CD4/lymphocyte values in blood samples are stable over a period of days, and distances in Malawi are relatively small, a national %CD4/lymphocyte service based on these three centers may be feasible.
